# Acknowledgment of Reviewers

**DOI:** 10.2188/jea.JE20200526

**Published:** 2020-12-05

The following scientists assisted the journal by reviewing manuscripts during the period November 1, 2019 to October 31, 2020.

We gratefully acknowledge their critical evaluation and assistance in selecting articles for publication.

**Table:** 

Abe, Sarah K.
Adachi, Hisashi
Ailes, Elizabeth
Akasaka, Hiroshi
Akter, Shamima
Al-Mohannadi, Abdulla S
Amagasa, Shiho
Anzai, Tatsuhiko
Aoki, Makoto
Araki, Atsuko
Asakura, Keiko
Ayesta, F. Javier
Baba, Sachiko
Babazono, Akira
Bae, Sanghyuk
Bradbury, Kathryn E
Budhathoki, Sanjeev
Burns, Jane
Cattaruzza, Maria Sofia
Cavalcanti, Yuri
Chavarro, JE
Choi, Kelvin
Chung, Seockhoon
Cooray, Upul
Demerath, Ellen
Doria, Alessandro
Drager, Luciano F.
Duncan, Laramie
Duncan, Laura J
Ebisu, Keita
Ewis, Ashraf
Forsum, Elisabet Katarina
Fu, Marcela
Fujihara, Kazuya
Fujii, Hitoshi
Fujii, Ryosuke
Fujita, Yuki
Fujiwara, Aya
Fujiwara, Takashi
Fujiwara, Takeo
Fujiyoshi, Akira
Fukazawa, Ryuji
Fukuda, Tatsuma
Fukui, Chie
Fukunaga, Ichiro
Fukushima, Wakaba
Furuta, Michiko
Gagnum, V
Gallus, Silvano
García-González, María Asunción
Garza, Cutberto
Gibo, Koichiro
Hachiya, Tsuyoshi
Hagimoto, Akiko
Hamada, Shota
Hara, Megumi
Harada, Kazuhiro
Haruyama, Yasuo
Hata, Jun
Hattori, Satoshi
Hayama-Terada, Mina
Hayashi, Katsuma
Henning, Margaret
Hidaka, Akihisa
Higashiyama, Aya
Hikichi, Hiroyuki
Hirata, Aya
Hirata, Takumi
Hirokawa, Kumi
Hisamatsu, Takashi
Ho, Manh Tuong
Honda, Takanori
Honjo, Satoshi
Hori, Ai
Hori, Megumi
Hornberger, Michael
Hosono, Satoyo
Ikehara, Satoyo
Imai, Yoshimichi
Inada, Haruhiko
Inoue, Shusaku
Inoue, Yosuke
Inoue-Choi, Maki
Ishihara, Junko
Ishikawa, Hirofumi
Ishimaru, Miho
Isumi, Aya
Ito, Hidemi
Ito, Yumi
Ito, Yuri
Iwamoto, Momoko
Iwasa, Hajime
Iwata, Osuke
Jackson, Dan
Jung, Sun Jae
Kachi, Yuko
Kadota, Aya
Kakamu, Takeyasu
Kakizaki, Masako
Kanamori, Satoru
Kang, Daehee
Kant, Ashima K.
Kashino, Ikuko
Katanoda, Kota
Kato, Tuguhiko
Kawabe, Kentaro
Kawakami, Ryoko
Kawasaki, Ryo
Kelly, Scout
Kidokoro, Tetsuhiro
Kikuchi, Hiroyuki
Kikuchi, Kazunori
Kishino, Hirohisa
Kitano, Naomi
Knudsen, Ann Kristin
Kobayashi, Masayuki
Kobayashi-Cuya, Kimi
Koriyama, Chihaya
Koyanagi, Yuriko
Kubota, Yasuhiko
Kurita, Satoshi
Kuriyama, Nagato
Kuroda, Yujiro
Kusama, Taro
Kuwabara, Kazuyo
Kuwahara, Keisuke
Kyoung Hwa, Ha
Lee, Hwi-Won
Lee, Yoon-Na
Leroy, Jef L
Li, Ya-Min
Lim, Min Kyung
Ling-Wei, Chen
Liu, Xiaoqiu
López-Suárez, Alejandro
Ma, Yunsheng
Martin, Jennifer L.
Martínez Sánchez, Jose María
Maruyama, Koutatsu
Matsumoto, Naomi
Matsuo, Keitaro
Matsuyama, Ryota
Matsuyama, Tasuku
Matsuyama, Yusuke
Matsuzaka, Masashi
Metoki, Hirohito
Michikawa, Takehiro
Minami, Ushio
Mitsuda, Naomi
Mittleman, Murray
Miyagawa, Naoko
Miyake, Yoshihiro
Mizusawa, Junki
Momma, Haruki
Monsivais, Pablo
Morisaki, Naho
Morita, Ayako
Moskal, Aurelie
Mossey, Peter
Motokawa, Keiko
Murakami, Keiko
Muraki, Isao
Nagao, Masanori
Nagata, Chisato
Nagatomi, Ryoichi
Naito, Mariko
Nakade, Makiko
Nakamura, Kazutoshi
Nakamura, Mieko
Nakamura, Yasuyuki
Nakano, Takashi
Nakata, Kayo
Nakata, Yoshio
Nakatochi, Masahiro
Nakaya, Tomoki
Nanri, Hinako
Narita, Akira
Nawa, Nobutoshi
Nemoto, Yuta
Ninomiya, Toshiharu
Nishi, Nobuo
Nishigori, Hidekazu
Nishimura, Kunihiro
Nishimura, Rimei
Nishino, Yoshikazu
Noma, Hisashi
Nordby, Kent
Nyman, Amy L
Oba, Koji
Oba, Shino
Ochi, Manami
Ogawa, Takahisa
Ohfuji, Satoko
Ohsawa, Masaki
Okada, Akira
Okada, Emiko
Okada, Rieko
Okada, Yohei
Okami, Yukiko
Okamoto, Etsuji
Okamoto, Shohei
Okamura, Tomonori
Okawa, Sumiyo
Okubo, Yoshiro
Onda, Yoshiko
Ono, Sachiko
Orui, Masatsugu
Orui, Masatsugu
Ota, Atsuhiko
Otani, Takahiro
Otsuka, Rei
Ozasa, Kotaro
Oze, Isao
Paratz, Elizabeth
Park, Boyoung
Perez-Escamilla, Rafael
Pladys, Adelaïde
Purohit, Bharathi M.
Raptou, Elena
Rasmussen, Svein
Rockette-Wagner, Bonny
Rodewald, Lance
Ryo, Okubo
Saeki, Keigo
Saijo, Yasuaki
Sairenchi, Toshimi
Saito, Eiko
Saito, Isao
Saito, Junko
Saito, Tami
Sakamoto, Haruka
Sakamoto, Naoko
Sakamoto, Tatsuaki
Sakurai, Masaru
Sasai, Hiroyuki
Sata, Mizuki
Sato, Kyoko
Sato, Tomoharu
Satoh, Atsushi
Satoh, Michihiro
Seino, Satoshi
Shibata, Yosuke
Shigematsu, Mika
Shimadzu, Hideyasu
Shimazu, Taichi
Shimizu, Yuji
Shinozaki, Hiromitsu
Shinozaki, Tomohiro
Shioda, Kayoko
Shitara, Kohei
Shypailo, Roman J.
Silva, Fernanda
Singh, Ambrish
Sirois, Fuschia
So, Hon-Cheong
Sone, Tomofumi
Subramanian, SV
Sugawara, Yumi
Sugiyama, Daisuke
Sugiyama, Hiromi
Sugiyama, kemmyo
Sulmont-Rossé, Claire
Surkan, Pamela
Suzuki, Etsuji
Suzuki, Harumitsu
Suzuki, Koya
Suzuki, Reiko
Tabuchi, Takahiro
Tachimori, Hisateru
Taguri, Masataka
Tajima, Naoko
Takahashi, Hirokazu
Takahashi, Kyo
Takahashi, Yoshimitsu
Takakura, Minoru
Takashima, Naoyuki
Takatorige, Toshio
Takeuchi, Ayano
Takeuchi, Kenji
Takeuchi, Yoshinori
Tanabe, Naohito
Tanaka, Hirokazu
Tanaka, Junko
Tanaka, Keiko
Tanaka, Keitaro
Tanaka, Sachiko
Tani, Yukako
Taniguchi, Yu
Tanikawa, Chizu
Tanno, Kozo
Tomata, Yasutake
Tsuboya, Toru
Tsujimoto, Takehiko
Tsukinoki, Rumi
Tsutsumimoto, Kota
Tsuzuki, Shinya
Uchida, Mitsuo
Ueda, Yutaka
Uehara, Ritei
Uemura, Kazuki
Ukawa, Shigekazu
Umesawa, Mitsumasa
Urushihara, Hisashi
Varghese, Nirosha Elsem
Volken, Thomas
Wada, Keiko
Wang, Chaochen
Wang, Zhenjie
Watanabe, Shuichiro
Yamada, Masaaki
Yamaguchi, Miwa
Yamaji, Taiki
Yamakita, Mitsuya
Yamamoto-Hanada, Kiwako
Yamana, Hayato
Yamaoka, Yui
Yamauchi, Takashi
Yamazaki, Shin
Yasuda, Yoshinari
Yokomichi, Hiroshi
Yokoyama, Yuri
Yoneoka, Daisuke
Yoshida, Daigo
Yoshida, Satomi
Yumoto, Tetsuya
Zaitsu, Masayoshi
Zaitsu, Takashi
Zhou, Fangjun

